# Using human centred design and human factors to support a rapid health information technology patient safety response

**DOI:** 10.1186/s12913-025-13293-5

**Published:** 2025-09-01

**Authors:** Selvana Awad, Rachel Begg, Thomas Loveday, Melissa T. Baysari

**Affiliations:** 1 The University of Sydney, Sydney, Australia; 2 eHealth NSW, Sydney, Australia

**Keywords:** Health information technology, Patient safety, Human factors

## Abstract

**Background:**

Human centred design (HCD) and human factors (HF) approaches can support the safe design and redesign of Health Information Technologies (HITs). Safety issues associated with HITs can occur due to complex sociotechnical and contextual factors which dynamically impact safety. An effective rapid HIT patient safety response could resolve issues before patient harm occurs.

**Aim:**

To describe, evaluate, and generate recommendations for optimising the application of HCD and HF methods by non-HF experts during a rapid statewide HIT patient safety response.

**Methods:**

In response to reported safety issues with a HIT, HCD and HF approaches were used by non-HF experts during site visits to understand the issues, contextual differences between sites and gather preliminary feedback on proposed redesign options to mitigate the issues. This quality improvement study involved two 45-minute focus groups with 7 staff who conducted the site visits to understand what worked well, what did not work well, and any lessons learnt relating to the application of HCD and HF informed data collection approaches during this patient safety response.

**Results:**

Outcomes from the site visits were reported to be variable. However, the overall data collection approach was considered effective in gathering useful information. Participants explained that key outcomes of the approach used included improved understanding of the issues and contributing contextual factors, effective engagement with sites and users, and increased team collaboration and job satisfaction among the data collection team. Participants identified factors that influenced the effectiveness of site visits including data collection approaches (e.g., individual vs group data collection), the circumstances on the day (e.g., time and availability of frontline staff), contextual factors (e.g., the nature of the unit/setting) and factors related to the data collection team (e.g., selection of team members involved). Recommendations have been provided to optimise future rapid HIT patient safety responses.

**Conclusion:**

Data collection approaches informed by HCD and HF methods are useful for understanding and addressing HIT safety concerns requiring rapid responses, even when applied by non-HF experts. Ideally, methods should be applied flexibly and involve seeking insights from frontline users in their own environments.

**Supplementary Information:**

The online version contains supplementary material available at 10.1186/s12913-025-13293-5.

## Background

Health information technologies (HITs), such as electronic medical records and electronic medication management systems, support care delivery by improving the quality, consistency, and accessibility of health information available to clinicians [1, 2]. While HIT has the potential to improve the safety and quality of care, unintended safety consequences can arise from its design, implementation and use [3, 4]. Despite best efforts to proactively design safe HIT using human centred design (HCD) and human factors (HF) approaches, robust testing, and safety assurance efforts prior to implementation, it is not always possible to fully pre-empt and design out hazards prior to HIT rollout [5].

Hospitals are complex adaptive systems consisting of interrelated system components and actors that dynamically interact with each other [6–8]. Safety is an emergent property of the interactions and relationships between these components [9–12] and the safety of healthcare organisations, including HIT, requires safety to be viewed through the lens of systems thinking [13]. While pre-implementation efforts may seek to ensure the design of interfaces and the configuration of functionality is as safe as possible, the safety of HIT is also influenced by complex sociotechnical factors such as the context of use, workflows, clinical practices, organisational policies, staffing, resources, digital health infrastructure, the quality of training provided, and other dynamic contextual factors at play within the local environments and ecosystems in which the technology is implemented [3, 13–15]. For this reason, it is not possible to predict or prevent all possible HIT-related safety events prior to implementation, particularly where a HIT system has been designed for state or national use, and therefore cannot cater for all possible contextual and dynamic variations within local environments that affect safety. In addition, as a system is exposed to more settings and used by more users, safety issues previously not identified may come to light [16]. For these reasons, safety concerns about a HIT can arise in a site even if other sites have implemented the same system and have not identified these safety concerns.

Limitations in testing conducted by system developers and/or implementing organizations may also contribute to the inability to fully predict safety issues prior to implementation. For example, often pre-implementation testing of HIT occurs in laboratory or controlled settings that do not adequately mimic real clinical environments where interruptions and distractions regularly occur [17–19]. While testing in these controlled settings can provide valuable insights and assist with identifying potential usability and safety issues associated with the HIT, it only tests the system within a static and linear environment where conditions are controlled. This type of testing does not reflect the complex adaptive nature of healthcare systems where changing conditions, including variations in clinical needs, caseloads and staffing may dynamically impact overall system safety including the safe use of HIT.

Testing strategies employed by organisations must also balance the robustness and comprehensiveness of testing, including the types and amount of testing conducted, with the amount of time and resources available to conduct testing within project constraints and timelines [20]. It has been recommended that this balance be guided by a risk-based approach i.e. the purpose of testing is not to prove that the system is risk-free, but rather to ensure that there has been sufficient testing to identify defects or hazards that have a higher probability of manifesting as harm and eliminating them or mitigating them to an acceptable risk level or So Far As Is Reasonably Practicable (SFAIRP) [20]. Despite best efforts to ensure testing robustness within project constraints, hazards and safety issues may still arise post implementation due to limitations in testing strategies and the inability to test all parts of the system under every possible circumstance that may present.

While it is possible to minimise HIT-related safety events through proactive safety and hazard identification methods, tools and processes during procurement, development and testing phases [5, 13], it is impossible to completely prevent the need for reactive safety management due to the inevitability of safety concerns and events, as well as unintended or unanticipated consequences [4, 5, 21, 22]. Safety concerns can arise during activities in the lead up to implementation, such as training, where the implementation of the system is being considered within local contextual factors, or after a system is implemented and a near miss or patient safety event occurs involving the system [2, 4, 21–25].

When safety concerns are identified post-implementation, effective and efficient reactive safety management processes can assist with understanding the nature of safety concerns, identifying system-related hazards and their causes, associated clinical risks and appropriate mitigations, including system redesign options [16, 26]. HCD is a development approach that aims to make systems usable through the application of human factors, and usability knowledge and techniques [27]. The discipline of human factors (or ergonomics) explores the interaction between humans and systems by understanding factors that facilitate the completion of work and aims to optimise human wellbeing and overall system performance [27, 28]. HCD and HF methods can be used to collect data around HIT safety concerns and their contributing factors from a sociotechnical perspective, and support the formulation of mitigations and recommendations including opportunities for redesign [29].

While some studies describe the use of such methods in the context of HIT design and redesign, limited evidence or guidance exists on how these approaches can be applied to support rapid reactive patient safety responses involving HIT [30]. Furthermore, despite the potential for HF methods to enhance system design, in our statewide organisation which is responsible for digital enablement across all public hospitals, limited HF expertise exists. This study is novel in that it describes and evaluates a statewide reactive patient safety response involving the practical application of HCD and HF methods by non-HF experts.

## Aim

This quality improvement study aimed to describe, evaluate, and generate recommendations for optimising the application of HCD and HF methods by non-HF experts during a rapid statewide HIT patient safety response.

## Methods

### Context

This project involved the review of safety concerns raised around an electronic medical record used in adult, paediatric and neonatal intensive care settings being rolled out or used across several hospitals in our state. The system was already live in a number of facilities, however as another facility was preparing for implementation (i.e. through training and go-live readiness activities), safety concerns were identified and escalated to the statewide HIT organisation overseeing the statewide rollout. A reactive patient safety response was activated to understand the nature of the safety concerns, and to develop mitigations. The response involved visits to sites which were preparing for implementation as well as sites which already had the system in place. A data collection approach informed by HCD and HF were used to understand issues, differences between sites and local contextual factors, and gather preliminary feedback on proposed redesign options to mitigate the issues. Outcomes from the site visit were used to inform working group meetings with clinical, technical, safety and quality, and design representation to agree on appropriate mitigations.

### Data collection approach

The approach used to collect data on the safety concerns involved a number of steps before, during and after the visits, as outlined in Table 1.

**Table 1:** Steps involved in the rapid HIT data collection approach undertaken

**1. Preparing for visits**
1.1 Initial understanding of the safety concerns
1.2 Definition of the scope and focus of the site visits including the specific functionalities associated with the safety concerns, alternate system design options (mitigations) to gather feedback on and stakeholders who would be consulted as part of the site visit e.g. clinical users, operational managers and executives, informatics staff, others
1.3 Identification of HCD and HF methods to be used, and development of a protocol to guide data collection
1.4 Identification of an appropriate pool of staff to conduct the site visits. Expertise of staff included: human centred design and human factors, safety and quality, and staff with technical understanding of system functionality. Data collection teams were formed based on appropriate skill set combinations (i.e. team members with understanding of the clinical or safety and quality aspects were paired with team members with understanding of the functionality) and deployed to different sites.
1.5 Identification of appropriate representative sites for visits
1.6 Communication with the selected sites on the purpose and scope of visits
**2. Conducting visits**
2.1 Site visit and data collection
**3. Post visit activities**
3.1 Data analysis and reporting of findings from the site visit
3.2 Dissemination of a site specific report to each site to allow sites to confirm the accuracy of information collected. Subsequent modification of reports were made where required.
3.3 Internal debrief between staff who collected data to compare findings between sites and synthesise recommendations to inform further working group discussions

### Protocol development including methods planned for use

Brief virtual meetings between the patient safety response lead in the statewide organisation (SA) and hospital-based subject matter experts (such as informaticians and clinicians) who raised the concerns were held to gain an initial understanding of the safety concerns. Guided by systems thinking, concerns raised underwent a preliminary analysis by the patient safety lead to understand their nature, and determine an approach to understand them further including potential methods that could be used. Overall, these interactions identified that the safety concerns were primarily focused on system usability issues and differences in clinical practice affecting safe use of the electronic medical record for medication related tasks. In particular, based on a description of the issues raised, they seemed to relate to (1) Task-technology fit issues i.e. mismatch between workflows used in practice for prescribing boluses and infusion rate changes, and workflows created by the functionality (2) Issues around how the system supports the visibility of context relevant information to support the administration of boluses and infusion rate changes (3) Potentially inappropriate system generated calculations and auto-population of total fluid intake from drug calculation weight (4) Differences in local clinical practices and policies around total fluid intake (5) Lack of intuitive design around ‘save’ and ‘save as’ buttons and what they mean (6) Potentially missing functionality to enable the documentation and witnessing of discarded controlled medications which is a policy requirement.

Given the issues related to system usability and safety, and contextual factors affecting safe use, a protocol was developed to support data collectors with understanding these usability issues further and seek feedback on redesign options. Two versions of the protocol were available; one for sites that had already implemented the system and a version for sites that had not yet implemented the system. The protocols were similar with respect to content however some of the questions were slightly different (e.g. for sites which had implemented the system, some questions were framed to gain an understanding of how the system was being used and whether the safety concerns identified were present). The protocol used for sites which had already implemented the system has been provided as an example in Appendix A.

Due to the limited presence of HF experts and qualitative researchers in our organisation, the response was led by a safety and quality expert with HF expertise and qualitative research skills (SA) and the protocol was primarily developed by the only other HF expert in the organisation (RB) to ensure that key usability and safety issues were explored appropriately. The rest of the data collection team consisted of personnel working in health informatics, configuration of the application, safety and quality, and change management. The data collection team had varying levels of skills in usability evaluation, user research and qualitative research methods more broadly. As such, the protocol (Appendix A) was developed as a user friendly tool to guide data collection in a consistent manner and the practical application of HCD and HF methods.

Prior to site visits, data collectors were provided with a brief overview of how the protocol should be used and were asked to consider of a range of usability evaluation and user research methods such as focus groups, in situ observations, individual semi-structured interviews, cognitive walkthroughs and usability testing (see Appendix A for scenarios) to gather information using the protocol based on what was feasible on the day. Thus, the protocol was designed to allow flexible use of these HCD and HF methods to gather required information on the specific areas of focus which were covered by the questions in the protocol.

The protocol (Appendix A), which was provided in a tabular format, included sections for data collectors to gather demographic information from participating users and stakeholders, and information on each issue identified from preliminary virtual meetings. For each issue, data collectors were provided with the following in the protocol: the priority level associated with the issue (e.g. high or medium) to assist users with prioritising data collection where time was limited, the relevant workflow or functionality, target users for that issue (e.g. medical or nursing staff), redesign options requiring feedback, prompting questions to guide data collection, and a section to document observations and user comments. These guiding questions could be applied flexibly irrespective of the method used e.g. focus groups, in situ observations, individual semi-structured interviews, cognitive walkthroughs and usability testing to gather information.

### Analysis and reporting of findings from site visits

Notes from the site visits were briefly reviewed and used to develop a reporting template by the patient safety response lead (SA) and HF expert (RB). All data collection teams were provided with this reporting template organised by key themes to allow for a consistent approach for analysing and reporting on findings from the site visits. Once each data collection team had discussed their findings through a de-brief session and populated the reporting templates to align with themes, the reports were shared with the sites visited to confirm and if needed, amend the content.

### Evaluation of lessons learnt

Staff involved in data collection were invited to participate in focus groups to evaluate lessons learnt via email, and therefore were purposively recruited. Focus groups were convened via videoconference to understand what worked well, what did not work well, and any lessons learnt relating to the application of the HF and HCD-informed data collection approach during this rapid HIT patient safety response. Questions used to guide the focus group discussions, which were developed for this study, are provided in Appendix B. Two 45-minute focus groups were run, one with 3 participants and the other with 4. The primary expertise of those who participated in the focus groups can be found in Table 2. All participants had clinical backgrounds (e.g. pharmacy, nursing and medical) except for the human factors specialist. The sessions were recorded and transcribed for data analysis. Transcripts from the focus groups were thematically analysed using an inductive approach by two independent coders. These researchers extracted codes related to factors that influenced the effectiveness of site visits and data collection, what worked well and what did not work well when preparing for and conducting site visits, and outcomes of the approach, and these were then grouped into themes. The two researchers came together to discuss identified themes and discrepancies in coding were discussed until consensus was achieved.

**Table 2:** Primary expertise of participants in focus groups who conducted data collection

Participant	Primary expertise
1	• Health informatics
2	• Configuration/application specialist
3	• Safety and quality
4	• Safety and quality
5	• Change management
6	• Health informatics
7	• Human factors

### Research reporting and ethics

This project was determined to be a quality improvement study, and therefore ethics approval was not required as per local guidelines [31]. It was waived by the St Vincent’s Hospital Human Research Ethics Committee. All participants provided informed consent, and the project was also compliant with the Declaration of Helsinki [32]. Focus groups transcripts were de-identified prior to analysis. The reporting of this project was guided by the Standards for Quality Improvement Reporting Excellence 2.0 guidelines [33].

## Results

### Sites visited and user types

Visits to representative intensive care units across neonatal, paediatric and adult settings were conducted across 4 sites. Overall, user types engaged included: 9 medical officers, 9 nurses, 2 pharmacists and 2 informatics professionals.

### HCD and HF methods used

Overall, participants reported using a range of usability evaluation and user research methods from the HCD and HF disciplines. Some data collection teams reported only being able to gather data in group settings through meetings with hospital representatives. Some reported having dedicated time with individual users in a quiet room to run semi-structured interviews, provide walkthroughs of the functionality and conduct light touch usability testing of redesign options. Some reported being able to observe workflows in the clinical environment. Data collection teams generally reported using one or more of the methods.

**Fig. 1:**
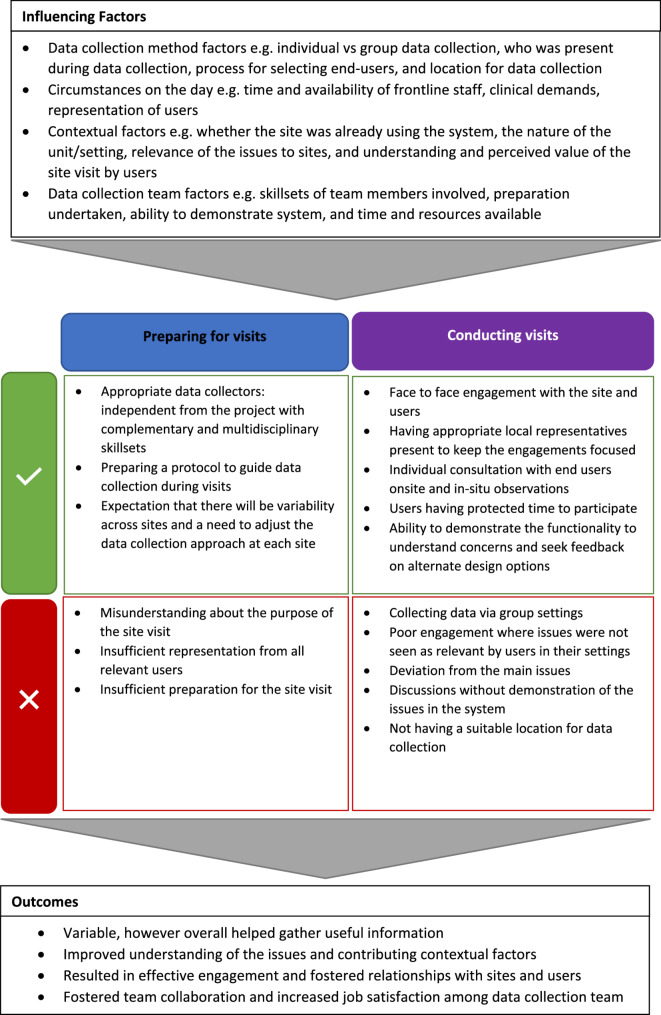
Influencing factors, what worked well and what did not work well when preparing for and conducting site visits, and reported outcomes

Results from the focus groups have been summarised in Fig. 1.

## Influencing factors, what worked well and what did not work well

### Skills of data collectors

A key preparatory aspect which was reported to work well during all site visits was ensuring that data collectors who visited each site reflected an appropriate and complementary combination of skillsets and backgrounds (Fig. 1). In particular, participants explained that it was important to pair data collectors who did not have an understanding of the system, such as those with HCD/HF or safety and quality backgrounds, with those who had an understanding of the system, and could explain and demonstrate the functionality: “*I think having those kind of multidisciplinary teams was a real strength of what we did and how it went”*.

The preparation of a protocol by a HF specialist to guide data collection during the visit was seen as instrumental in preparing all data collectors for the visits, ensuring that data collection remained focused on usability issues, and feedback on alternate designs was obtained: “*I can say for certain*,* though*,* what really*,* really helped was the [protocol]… the layout of the interviews*,* how it was structured*,* I found that was super helpful*,* especially if I was stuck on an answer it was it helped me bring the conversation back to the point rather than allowing that person to deviate from the conversation”.* Participants also commented that due to the variability in experience among the data collection team, some members could have benefited from a pre-visit briefing session to enable data collection upskilling and clear *“delineation of roles”* among the data collectors during the visit, for example, facilitators vs. note takers. It was acknowledged however that due to the rapid nature of the response, this was not possible. With respect to the protocol itself, participants suggested that in future, a digital version should be considered to support data collection.

While the protocol served as a support tool for all data collectors, participants commented that data collectors who had prior exposure to HCD and HF demonstrated valuable skills in adapting the protocol in real time to elicit nuanced information from end users using open-ended questioning and probing techniques: *“…because I know the system…sometimes I didn’t ask a question in a question way*,* because I was anticipating answers… I found it really useful to have one of you guys who are good at [human centred design] wording the questions a lot better to not necessarily point to the answer*,* which is modifying what I did sometimes. Because I know this is a leading question… So I think having that dual*,* the two different knowledge bases actually really worked well for us*,* because we did tend to split up in groups of one [person without knowledge of the system and one person with knowledge of the system]…and I think that worked really well”.*

### Preparation for site visits

Participants reported that where data collection teams had conducted sufficient preparation for the site visit, for example, by ensuring the site was fully aware of the purpose of the site visit and all relevant personnel at the site were adequately briefed, user engagement tended to be more effective. This supported the collection of high-quality information. Participants explained that where users were not aware of or misunderstood the intent of the visit, for example, if they assumed that the visit was an opportunity to provide feedback about any aspect of the system rather than the specific issues related to the patient safety response, users were less engaged and interested in participating in data collection: *“But I did know that when we went to [particular setting]*,* to ask the same questions*,* they were less interested because they didn’t find [the issues] as concerning*,* and therefore wanted to use their time to suggest some other enhancements that could be made to make the application better”*.

### Other factors that affected data collection

Other contextual factors that were reported to affect user engagement included whether the site was already using the system, the nature of the unit or setting, and the perceived relevance of the issues to the sites and units.

Participants reported that the data collection approach for each site varied and was required to be adapted based on the circumstances on the day. Participants explained that the site visits involved flexible and adaptable application of a combination of all the methods rather than purist application of any specific method alone. Expecting that there would be variability and a need to adapt was seen as a helpful preparation strategy for the site visit: *“We had that adaptability and expectation that…we may not get everything that we want*,* which is exactly what happened”.* For example, where there were high clinical demands on units or users did not have protected time to be involved, participants said that users were not as readily available to participate in data collection. Focus group participants mentioned that in some cases, it was difficult to achieve representation from all relevant users. Overall, participants explained that data collection teams were required to adapt their approaches to gather as much data as possible within the constraints and challenges which presented on the day: “*I think in those situations*,* you sometimes just have to make do the best you can with what you’ve got right?”*

Data collection was seen as more effective when the system and relevant functionality could be demonstrated to support feedback gathering, as opposed to when demonstrations did not occur. In addition, focus groups participants reported that collecting data from individual users in appropriate locations (e.g., in the clinical environment) yielded useful and potentially more accurate information compared to collecting data in group settings, for example via focus groups, which could be influenced by groupthink: “*If they’re in groups…they tend to choose their words. But when you talk with individual users*,* that’s when the actual workflow comes out and you hear about the workarounds … if it’s a focus group*,* that sways it a bit*,* because there’s groupthink*,* but with actual users on the floor*,* showing us what they do*,* I think that’s where we actually get the context of use more”.* Participants reported that in some cases, the presence of senior staff in a group setting may have influenced what users were willing to share. However, at other times, having appropriate local representatives present to keep the discussion focused was seen as helpful.

### Continuity of data collectors

Participants said that having the same data collection team at each site would have been helpful to enable easier comparative analysis post-visits: “*It would have been great if the same people could have gone to every site so the same people with the same expertise*,* asking the same questions in the same way”.* The available time and resources of the data collectors also influenced the amount of preparation that could be undertaken before the site visits, the timeliness of data collection and analysis, and their ability to be involved in ongoing working group meetings following site visits. Data collectors who remained involved from response initiation to completion commented that winding back the involvement of other data collection team members after the site visits and initial working group meetings impacted continuity and disrupted the rapport that had been built with sites and users throughout the response: “*So I just think potentially*,* if we had remained as a group … we might have been able to wrap it up a bit earlier*”.

### Overall outcomes of the approach

While there was some variation in how site visits were conducted and their outcomes, overall, participants found the HCD and HF approach positive, appropriate, and helpful in gathering information about the safety concerns. Participants reported that the site visits assisted with understanding which of the safety issues affected sites and settings, contextual factors such as *unit specific* practices that contributed to the potential safety issues being present, and in some cases helped elicit information that was not previously known through other forms of engagement such as virtual meetings: “*Some of the things are very unit specific*,* which is also great data to collect*,* because that’s why [they’re] having such issues because no one else is doing this”*.

In addition to enabling data collection and enhancing understanding of the concerns, focus group participants highlighted that a key benefit from the site visits was effective engagement with clinicians and other staff at the sites which made them feel heard and fostered interpersonal relationships: “*I…think that definitely the site visits were appropriate …there’s a special function of face-to-face engagement that just can’t be done over electronic means*,* whether it’s email or Teams*,* so I think we definitely did need to be there”.* Participants reported that fostering these relationships and creating rapport through the site visits would likely contribute to the success of future engagements with the organisations.

The data collectors, who came from different teams within the organisation and had varied skillsets, also reported that the site visits fostered team collaboration among themselves and increased appreciation for each other’s skills and roles, enhanced their job satisfaction, and provided a valuable learning experience: “*I valued what I learned and what I got out of the experience”.*

### Key recommendations

Table 3 summarises key recommendations provided by participants (data collectors) during focus groups after the site visits around the use of HCD and HF approaches, to inform future rapid HIT patient safety responses.

**Table 3:** Key recommendations provided by participants (data collectors) during post site visit focus groups

Proactive vs. Reactive	Category	Recommendation
***Proactive *** **Safety Management**	**Preventative measures**	• Ensure proactive hazard identification, adequate testing and HCD/HF methods are used to inform the design and identify safety issues prior to deployment to minimise reactive responses post implementation • Conduct site visits early on post implementation and regularly thereafter to ensure ongoing surveillance and management of safety issues as they arise
***Reactive *** **Rapid Patient Safety Responses**	**Preparing for visits**	• Identify sites which should be visited. Where possible, visit all sites affected and where this is not possible, identify a representative sample to cover clinical settings where the system is being used • Work with site visit representatives to: o Ensure the purpose of the site visit is clear and users are prepared before visits o Ensure that communication about the rationale for the site visits/data collection has a patient safety focus o Discuss end user recruitments and strategies for promoting end user engagement o Ensure appropriate locations for data collections o Identify and implement strategies to mitigate the risk of users not sharing information due to the presence of senior colleagues o Identify and cater for logistics and other site specific considerations to be aware of on the day • Consider different ways of collecting data and ensure the approach is adaptable e.g., usability testing of functionality under review, conduct discussions virtually and face to face, surveys • Prepare a protocol to guide data collection • Identify appropriate combination of skill sets for data collectors and where possible, send the same data collection team to each site • Assign clear roles for data collectors, and ensure they are adequately skilled and prepared for site visits
	**Conducting visits**	• Consider digital options to support data collection where possible • Ensure agility and adaptation of data collection approach where required • Where possible, conduct data collection via individual consultations in situ, supported by system demonstrations
	**Post visit activities**	• Conduct de-briefs for data collectors to discuss and synthesise findings

## Discussion

This quality improvement study described, evaluated and generated recommendations about optimising a HF and HCD-informed data collection approach during a rapid HIT patient safety response. Reactive patient safety responses may sometimes be viewed as negative events despite their inevitability; however, as our results showed, they provide opportunities for system optimisation and continuous improvement efforts. Our evaluation of the approaches used in this rapid patient safety response revealed that outcomes of the approach were variable, however, overall site visits were considered helpful in understanding safety issues. Furthermore, several factors influenced the effectiveness of site visits including how data were collected (for example, individual vs. group data collection), circumstances on the day, contextual factors and factors that related to the data collection team.

While methods such as Root Cause Analysis and the London Protocol are used to retrospectively investigate and understand contributing factors related to patient safety events including those involving health information technologies, further research is required to understand how underpinning methods of engaging users for feedback and understanding the context of use can support these safety approaches [30]. The HF and HCD-informed data collection approach used in this study assisted with identifying specific contextual factors that contributed to safety concerns by gathering information from users and relevant stakeholders. Although this study involved understanding and addressing safety concerns identified during pre-implementation activities, the approach applied may be useful in supporting patient safety responses where a safety event has occurred (e.g. to feed into Root Cause Analyses). As understanding a system’s context of use is essential during initial design, iteration and continuous improvement phases to ensure that systems are safe and usable within specific contexts [27], where patient safety concerns arise, as demonstrated by this study, efforts should be made to understand the broader system and contextual factors that may be contributing to these concerns. This is because the first step of the HCD process which aims to develop usable systems is to understand and specify the context of use [27]. Context of use refers to ‘the users, goals, tasks, resources, and the technical, physical and social, cultural and organisational environments in which a system, product or service is used’ [27]. As usability and safety are emergent properties of the interactions and relationships between different system components and actors, the same HIT system can have differing usability and safety profiles within different contexts of use [9–12, 27].

As demonstrated in this project, HCD and HF approaches which involve engaging with frontline users in their own environments can effectively support the collection of data about safety concerns within local contexts and generation of user insights as part of rapid HIT patient safety responses. Major benefits reported in this study included the gathering of useful information and improved understanding of the issues and contributing contextual factors, but also some unanticipated benefits including effective engagement with sites and users, and increased team collaboration and job satisfaction among the data collection team. Whilst it is possible to gather information through remote means without face to face user engagement in clinical environments, for example through remote meetings, significant value was found in speaking to representative frontline users in their own environments as this enabled the identification of insights that may not be possible through remote options (for example, where factors associated with the live environment needed to be pointed out or demonstrated) and the building of rapport. Similarly, other studies have found that while remote options may improve access to participation by providing flexibility, they compromise opportunities for rapport and trust building in relationships, and active engagement afforded by face to face options [34, 35].

A key learning was that data collection needs to be adapted based on circumstances on the day. While engaging users in their own environments demonstrates commitment to understanding context of use, this study found that there were challenges with applying HCD and HF methods involving representative users in busy clinical environments. These findings however are not unique to the rapid HIT patient safety response context. Other studies have reported similar challenges with user involvement in HIT development such as organisational and cultural factors, lack of time for users and project teams, and challenges with accessing representative clinicians due to workloads and other factors [36–40]. Noting these challenges, this study and others highlight the need for strategies to support clinician involvement, adaptable application of methods and employing a combination of complementary methods based on aims/needs, feasibility, resources and timelines [30, 36, 38].

All participants found the participation of a HF expert to be very useful particularly with respect to designing the data collection protocol. While HF methods have significant potential to add value to HIT design and redesign processes including rapid patient safety responses [41], the lack of a human factors workforce in healthcare, including HIT, limits this potential [42]. Furthermore, human factors analysis methods can sometimes be complex to apply, potentially time-consuming and require a more advanced skillset [43]. As seen in this study, there may be an argument for reserving the designing and planning of HF approaches based on appropriate method combinations for HF practitioners, while providing a baseline level of upskilling to non-HF practitioners such as HIT designers or informatics staff who may be drawn upon to support data collection [44–47].

This study identified that data collectors could have been better prepared for the visits through briefing sessions that provided upskilling and clarified their roles during the visit. While it was not possible to provide such briefing sessions due to the rapid nature of the response, future rapid patient responses may benefit from proactive upskilling initiatives efforts that define basic and advanced competencies. This could assist with ensuring that there is an appropriately skilled pool of individuals available for deployment during rapid HIT patient safety responses as part of business preparedness efforts. For example, training in data collection skills such as open-ended questioning techniques and observations may be useful as they serve as the basis of many HF methods. In addition, training data collectors in how to adapt data collection based on circumstances may be useful as purist application of methods is often not feasible. Proactive, surveillance focused safety site visits may be useful in proactively identifying safety issues as well as providing opportunities for data collectors to practise data collection skills that underpin HF methods. Finally, as highlighted in this quality improvement study, the availability of human factors based protocols, tools and templates that can be quickly adapted and used to support data collection efforts in the context of rapid patient safety responses may also be beneficial.

While a major strength of this quality improvement study is that it is based on a real world rapid HIT patient safety response, it also has several limitations. In particular, the evaluation relied on the use of focus groups which were useful in generating insights however could have been affected by groupthink. Furthermore, the study did not employ other forms of evaluation to quantify the impact of the HF approach on the patient safety response. Although findings from this study suggest that the methods used (observations, interviews, focus groups and usability testing) to inform the data collection protocol enabled sufficient collection of information about the safety concerns and were guided by systems thinking, they did not factor in data collection around adaptability. There may be future opportunities to explore the application of other systems thinking HF methods that focus on understanding how adaptation impacts safety within complex sociotechnical systems such as Cognitive Work Analysis (CWA), Critical Decision Method (CDM), Systems Theoretic Accident Modelling and Processes (STAMP), Functional Resonance Analysis Method (FRAM) and the Event Analysis for Systemic Teamwork (EAST) method [37]. Finally, future opportunities could also seek to structure findings using frameworks such as the Systems Engineering Initiative for Patient Safety (SEIPS).

## Conclusions

HIT patient safety concerns or events such as unintended consequences are inevitable due to many sociotechnical and contextual factors which dynamically impact safety. Where safety concerns arise, reactive responses to understand and address the issues should be supported by HF and HCD methods involving end users in their environments that aim to understand the context of use and other contributing factors. A number of strategies can be implemented to ensure that site visits are effective such as development of a HF/HCD-informed approach and data collection protocol by a HF specialist, selecting the appropriate combination of skillsets within data collection teams, ensuring adaptability and flexibility, and preparing sites for the visits.

## Supplementary Information

Supplementary Material 1

Supplementary Material 2

## Data Availability

The data generated and analysed during the current study are available from the corresponding author on reasonable request.

## Abbreviations

CDM: Critical Decision Method
CWA: Cognitive Work Analysis
EAST: Event Analysis for Systemic Teamwork
FRAM: Functional Resonance Analysis Method
HCD: Human centred design
HF: Human factors
HIT: Health information technology
STAMP: Systems Theoretic Accident Modelling and Processes
