# Supplementary material for: Using human centred design and human factors to support a rapid health information technology patient safety response
Source: BMC Health Serv Res. 2025 Sep 1;25:1169. doi: 10.1186/s12913-025-13293-5 (PMC12403536; doi:10.1186/s12913-025-13293-5)
Supplement: Supplementary file 1 — Supplementary Material 1 [file 12913_2025_13293_MOESM1_ESM.docx]

**Appendix A – Protocol for sites with the system**

This protocol contains guiding questions which can be applied flexibly irrespective of the method used e.g. focus groups, in situ observations, individual semi-structured interviews, cognitive walkthroughs and usability testing to gather information.

**Site:** (e.g., hospital name)

**Location:** (Adult / Child)

**Date:**

**Name of Tester:**

**General Background:**

What is your role?

How long have you been working in intensive care at this hospital?

How long have you been using the system?

On a scale of 1 to 10, with 1 being beginner and 10 being expert, what would you rate your understanding and use of the system?

**Interview / Observation of Current System:**

| Issue | Workflow / functionality | Users | Prompts | Observations & User Comments |
| --- | --- | --- | --- | --- |
| 1 – High priority | Prescription of infusion boluses & rate changes  **Functions**  Prescribing; Modify function; Progress notes usage | Medical Officers | Can you walk me through how you **prescribe** infusion boluses to be administered from a running infusion?  Do you **use progress notes** when prescribing infusion boluses?  Can you walk me through how you would **document** you want to increase the rate of a running infusion for your patient? E.g. dobutamine  Do you use the modify function for medication orders? If yes, what for? If no, why/what do you do instead? |  |
| 1 – High priority | Administration of infusion boluses & rate changes  **Functions** Rate change functionality, Bolus administration functionality, Second person check documentation | Nurses  Pharmacists (1 question) | What usually happens if a doctor wants the **rate of an infusion to change** for your patient (e.g., from 10mL/hr to 5ml/hr)? Is there a place you check in the system to see what the doctor wants? How do you document a second person check if required for a rate change?  What usually happens if a doctor wants a **bolus** **of 10ml to be administered from a running infusion** for your patient? Is there a place you check in the system to see what the doctor wants?  [Pharmacist] How do you review infusions when trying to understand any rate changes and bolus doses? |  |
| 2 – High priority | [Neonatal] Calculation of total fluid intake  **Functions** Total fluid intake, nursing re-calculating TFI and where it is documented | Medical Officers; Nurses | How do you calculate Total Fluid Intake?  What do you think of the total fluid intake automatically calculating from the drug calculation weight? Do you have any concerns?  Are there scenarios where the total fluid intake should be different to the one from the one automatically generated from the drug calculation weight? How often does this occur? What would you do in these instances?  Do you have any suggestions on how the system could be changed to improve how the total fluid intake works? |  |
| 3 – Medium priority | “save” & “save as” functionality  **Functions** Save functionality, Save as functionality | All clinicians | Could you tell me what you think the “save” button does for documentation such as progress notes?  [Neonatal] Could you tell me what you think the difference is between the “save” and “sign as” buttons?  What do you think of the “save” and “sign as” functionality in the system? Any frustrations or concerns?  What do you think the impact would be if clinicians were to **sign every ‘progress note’** with their login credentials regardless of whether it is a new note or editing an older note? |  |
| 4 - Medium priority | Single user able to document and witness discard of S8/S4D medications  **Functions** Discard S4D/S8 Medications | Nurses  Pharmacists (1 question) | How would you document 10 mL from a morphine syringe was discarded because it was not required? Do you usually use the form attached to the medication order or the general one at the bottom of the screen?  What are your thoughts on the S4D/S8 discard form? Any thoughts on the second person witness workflow with the S4D/S8 discard form?  [Pharmacist] How would you review if excess S8 medication in an infusion was discarded in the system? Is this something you would need to do? |  |

**User Testing of Alternate Design Options:**

| Test Patient ID | Workflow / functionality | Users | Testing Instructions | Prompts | Observations & User Comments |
| --- | --- | --- | --- | --- | --- |
| PrescribingOne, Usability  PrescribingTwo, Usability  PrescribingThree, Usability  PrescribingFour, Usability  PrescribingFive, Usability  PrescribingSix, Usability | **Issue 1** Prescription of infusion boluses & rate changes  **High priority**  **Function**  Modify function, bolus ordering | Medical Officers | Please **modify the rate** of an already running midazolam infusion from 1 microg/Kg/minute to 2 microg/Kg/minute. | What are your thoughts on using the modify rate workflow?  How does it compare to what you are currently doing? |  |
|  |  |  | [Optional in Neonatal] Please **prescribe a separate bolus midazolam medication order** of 50 microg/kg to be run from an already running infusion  (In Adult settings - expert to walk through the prescribing of a bolus because it is a Neonatal layout: Sedation-IV Intermittent button. Say, prescribing a bolus is how you would normally prescribe any once only medication order) | What are your thoughts on prescribing a separate bolus medication order?  How does it compare to what you are currently doing? |  |
| NursingOne, Usability  NursingTwo, Usability  NursingThree, Usability  NursingFour, Usability  NursingFive, Usability  NursingSix, Usability | **Issue 1**  Administration of infusion boluses & rate changes  **High priority**  **Function**  Dose action review | Nurses | You have been verbally notified to change the rate of an already running midazolam infusion. This has been entered into the system directly into the infusion order. Please use the system to review the details of the request to change the rate.  Now please use the system to document the change in rate of the infusion including the use of the new form we have attached to the infusion dose. Assume you need to have a second person witness for the rate change. We will the provide the login details of the second person witness. | What are your thoughts on using the modify rate workflow?  How does it compare to what you are currently doing? |  |
|  |  |  | [Optional in Neonatal] You have been verbally notified to administer a bolus from an already running infusion. This has been entered into the system as a separate medication order. Please use the system to review the details of the request to administer a bolus.  Now please use the system to administer the bolus. Assume you need to have a second person witness for the bolus administration. We will the provide the login details of the second person witness. | What are your thoughts on prescribing a separate bolus medication order?  How does it compare to what you are currently doing? |  |

**Test Scenarios (if conducting usability testing)**

Medical officer scenario

For the Prescribing test patients,

- Prescribe a Midazolam seizure loading+maintenance order starting at 1 microg/kg/minute.
- Remove the loading once only order
- Validate the dose through the dose and task list
- Mark in error and Undo validate any older orders and administered doses from previous sessions

Nursing scenario

For the Nursing test patients,

- Prescribe a Midazolam seizure loading+maintenance order starting at 1 microg/kg/minute **starting at least 1 hour before current time**
- Validate the dose through the dose and task list
- Modify the prescribed midazolam order from the dose rate of 1 microg/kg/minute to **2 microg/kg/minute**
- Prescribe a ONCE ONLY midazolam 50 microg/kg once only bolus (sedation-IV intermittent button)
- Mark in error and Undo validate any older orders and administered doses from previous sessions
