# Supplementary material for: Using human centred design and human factors to support a rapid health information technology patient safety response
Source: BMC Health Serv Res. 2025 Sep 1;25:1169. doi: 10.1186/s12913-025-13293-5 (PMC12403536; doi:10.1186/s12913-025-13293-5)
Supplement: Supplementary file 2 — Supplementary Material 2 [file 12913_2025_13293_MOESM2_ESM.docx]

**Appendix B – Questions used during focus groups**

1. Site visit discussion
   1. What were the key goals / outcomes you aimed to achieve through the site visits? Did you feel that site visits were appropriate for understanding the raised concerns?
   2. How did the site visits contribute to achieving these outcomes? How helpful were they? Why/why not?
   3. What methodologies / approaches / tools did you employ to collect information on safety concerns related to the solution? Did this vary between sites?
   4. What factors influenced the data collection approach during each site visit?
2. What worked well
   1. What aspects of the design data collection approaches worked well during the patient safety response?
   2. Can you outline any clear benefits from the data collection approach achieved during the site visit? How did the site visits influence the overall patient safety response? How effective was the data collection approach in gathering the required information?
   3. Can you share specific examples of how the HCD and HF design data collection approaches helped uncover important insights or identify safety concerns?
   4. In what ways did the HCD and HF approaches contribute to the understanding of the safety concerns in the ICU context?
3. What did not work well
   1. What aspects of the design data collection approaches did not work well during the patient safety response?
   2. What were the challenges encountered while implementing the design data collection approaches?
   3. How were these challenges addressed?
4. Key lessons learnt
   1. What were your main lessons learnt and insights from the application of HCD and HF design data collection approaches in this patient safety response? Please share any expected and unexpected findings/reflections.
   2. Looking back, what changes or improvements would you suggest for the design data collection approaches used during the patient safety response?
   3. How can these lessons inform future initiatives?
